# Advanced abdominal pregnancy, with live fetus and severe preeclampsia, case report

**DOI:** 10.1186/s12884-017-1437-y

**Published:** 2017-07-26

**Authors:** Fekade Getachew Hailu, Getnet Tesfaye Yihunie, Ahmed Amdihun Essa, Walelign kindie Tsega

**Affiliations:** 1 Mariestops International Ethiopia, Bahirdar, Ethiopia; 2 Bahirdar University, Bahirdar, Ethiopia

**Keywords:** Advanced abdominal pregnancy, Ectopic pregnancy, Severe preeclampsia

## Abstract

**Background:**

Abdominal pregnancy may account for up to 1.4% of all ectopic pregnancies. The incidence of abdominal pregnancy differs in various literatures and ranges between 1:10,000 pregnancies to 1:30, 000 pregnancies. The clinical symptoms of an uncomplicated abdominal pregnancy are unspecific. There are reports of maternal and fetal survival from advanced abdominal pregnancies.

**Case presentation:**

Our case was a 26 years old gravida 4, para 3 (2 alive, one early neonatal death) woman. She presented to Felegehiwot Referal Hospital with a principal complaint of vomiting, epigastric pain, headache, and blurring of vision. Emergency cesarean delivery was decided with the impression of bicornuate uterus with intrauterine pregnancy, intrauterine growth restriction and sever preeclampsia.it was found to be advanced abdominal pregnancy. Placenta was removed and pack was used to control bleeding. Both the mother and neonate were discharged in a good condition.

**Conclusion:**

Abdominal pregnancy with live fetus is an extremely rare condition and requires a high index of suspicion. Endometrial cavity may not be required for development of severe preeclampsia and packing is effective in controlling bleeding in selected cases.

## Background

Abdominal pregnancy may account for up to 1.4% of all ectopic pregnancies [1]. The incidence of abdominal pregnancy differs in various literatures and ranges between 1:10,000 pregnancies to 1:30, 000 pregnancies [1]. The incidence is high in women of developing nations [2]. This may be due to low socioeconomic status, high rate of pelvic inflammatory disease or pelvic infection, history of infertility, tubal sterilization, tubal reconstruction surgery, pregnancy with intra uterine device [2]. Compared to tubal and intrauterine pregnancies the risk of dying from abdominal pregnancy is high. It is 7.7 times higher than tubal pregnancy and 90 times greater than an intrauterine pregnancy [2]. The maternal mortality may range from 0.5% to18% and perinatal mortality rate is 40–95% [2].

It can be either a primary or secondary. Primary peritoneal implantation is rare, and proposed criteria for its diagnosis include the following: normal tubes and ovaries, absence of uteroplacental fistula, and sufficiently early diagnosis to exclude the possibility of secondary implantation [3]. The secondary type is the commonest and it is commonly following ruptured tubal pregnancies [3]. The definition of abdominal pregnancy excludes Ovarian, tubal and intraligamentary pregnancies [3].

The clinical manifestations of an uncomplicated abdominal pregnancy are unspecific [4]. The most frequently encountered includes: non-labor typically persistent abdominal or suprapubic pain (100%), no delay in menstruation, bloody vaginal discharge, gastrointestinal symptoms (70%), painful fetal movements (40%), malaise (40%), and altered bowel movements [4].

Even if the association has rarely been described the incidence of pre-eclampsia would be expected to be high in such patients [5].

We report here a case of advanced abdominal pregnancy with severe preeclampsia. Both the mother and her baby were discharged safely.

## Case presentation

Our case was a 26 yrs. old gravida 4, para 3 (2 alive, one early neonatal death) woman. The gestational age from reliable last normal menstrual period was 37 weeks and 2 days. She had regular antenatal care follow up at local health center. She presented to Felegehiwot Referral Hospital with a principal compliant of vomiting, epigastric pain, headache, and blurring of vision. She also had history of nausea, vomiting, and abdominal pain 2 months prior to her arrival to the hospital. She had no urinary complaints. She had no history of hypertension or diabetes mellitus. She was referred from nearby general hospital after being given magnesium sulfate.

**Fig. 1:**
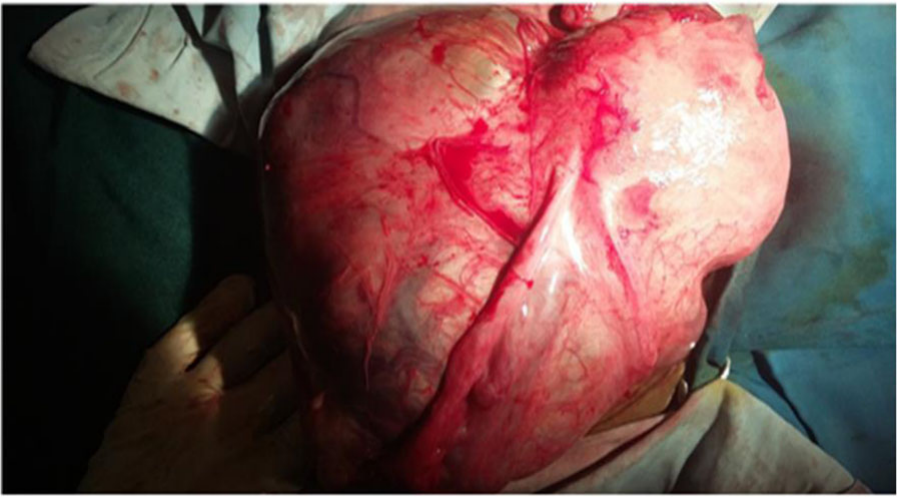
Intact amniotic membrane

On examinations, her blood pressure was 150/100 mmHg.Her respiratory rate, pulse rate and temperature were 22/min, 112/min, and 36.8 °C respectively. On abdominal examination, there was a 6 cm by 4 cm mass on left lower quadrant. The mass was smooth, firm and non-tender. Her symphysis fundal height was 28 weeks sized gravid uterus, with longitudinal lie and cephalic presentation. The fetal heart rate was 132 beats per minute and there were no uterine contractions. On vaginal examination the cervix was closed and uneffaced. The presenting part was not accessible and Head was palpable in the posterior cul-de-sac. On ultrasonography examination, Liver looks normal. There was mild right hydronephrosis. There was a singleton intrauterine viable pregnancy, and gestational age was 30 weeks by ultrasound estimation. The FL to AC ratio was 24.2. The Placenta was on the body of uterus anteriorly. There was no measurable amniotic fluid. There was separate empty uterus sharing same myometrium with existing pregnant uterus. Her preoperative hemoglobin was 11.6 mg/dl and platelet count was with in normal limit. Her urine albumin was +2.the renal and liver function tests were with in normal limits. Emergency cesarean delivery was decided with the impression of bicornuate uterus with intrauterine pregnancy, intrauterine growth restriction and sever preeclampsia. Upon opening her abdomen and entering the peritoneum, there was no heamoperitoneum and the fetus was seen in an intact amniotic sac (Fig. 1).

**Fig. 2:**
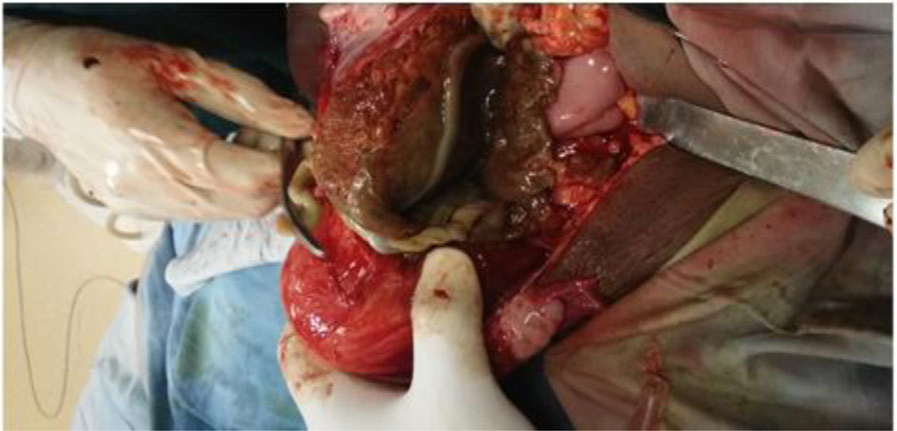
Placental site after the baby delivered

**Fig. 3:**
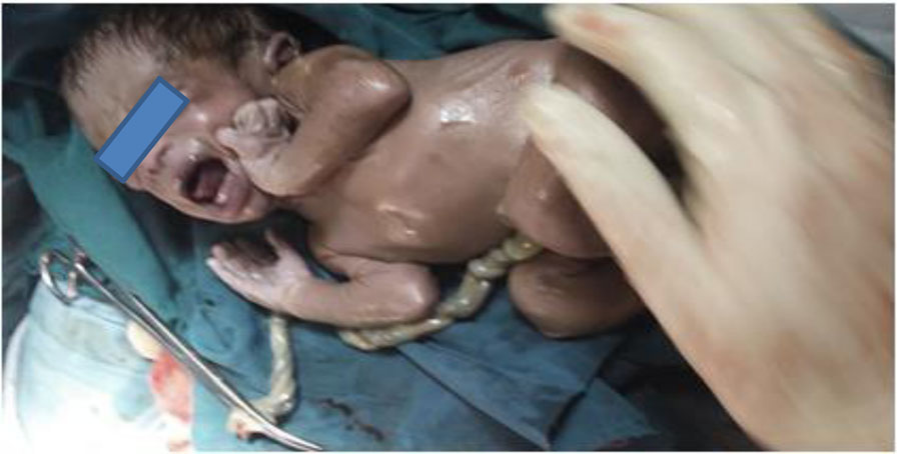
Baby immediately after delivery

The amniotic sac was opened and a live male neonate was delivered weighing 1.8 kg.The Apgar score was 7&8 at 1st and 5th minutes respectively. Both the fallopian tubes and uterus were normal looking. There was no rupture or fistula seen on the uterus. The ovaries were normal and the placenta was implanted on posterior aspect of the uterus and the right broad ligament. There was no bowel or major vessel attachment. The placenta was removed by detaching it from fundal part of the uterus down to the cul-de-sac. The placental attachment site was bleeding and it was difficult to arrest bleeding just by compression. We used 3 packs and kept it in place by suturing the fetal membrane remnants and omentum with the broad ligament. After this, the bleeding stopped and the patient left the operating room with stable vital signs (Fig. 2). She was transfused with 2 units of cross matched blood. She was put on Ceftriaxone and metronidazole. The packs were removed by doing laparotomy after 24 h. The pack was soaked with blood but no collection or surface bleeders. Patient kept on intravenous antibiotics for 4 days. On the 5th postoperative day the antibiotics changed to PO and she was discharged home with her neonate. She returned back to the hospital after 3 months. She was normotensive and her baby was 5.1 kg and healthy (Fig. 3).

## Discussion and conclusions

An abdominal pregnancy is a rare type of ectopic pregnancy, which may account for about 1% of all ectopic pregnancies [1]. It is associated with high maternal and perinatal mortality. A review of literatures from 2008 to 2013 showed that 38 cases of an AAP resulting in a live birth were identified from 16 countries [6].

Abdominal pregnancy could be either primary or secondary [6, 7]. The latter is the commonest type. To consider abdominal pregnancy as primary, the pregnancy must meet the three criteria [8]. The first is both tubes and ovaries must be in normal condition with no evidence of recent or remote injury [8]. The second is no evidence of utero-peritoneal fistula should be found [8]. The third is pregnancy must be related exclusively to the peritoneal surface and be early enough to eliminate the possibility that it is a secondary implantation following a primary implantation in the tube [8]. In our case both the ovaries and tubes were normal in appearance and we didn’t identify any utero-peritoneal fistula. But the third criterion is not clearly met. In the case of primary abdominal pregnancy placenta sits on the intraabdominal organs generally the bowel, mesentery, or the peritoneum [8, 9]. So we assume that it may be a case of secondary abdominal pregnancy.

Commonly abdominal pregnancy is easily missed and diagnosed after substantial emergency bleeding. This may be caused by less vascularized placenta, a weak gestational sac, and the lack of protection of the myometrium [7]. There are no widely accepted diagnostic criteria for abdominal pregnancies and the current diagnostic criteria for primary abdominal pregnancy are based on Studdiford standards [6, 7]. Patients with abdominal pregnancy typically have persistent abdominal and/or gastrointestinal symptoms during their pregnancy [10]. This is also true in our case.

Abdominal pregnancy often leads to early spontaneous separation of the placenta from implantation site, causing abdominal bleeding. In rare cases, the pregnancy can develop to late stages like in our case [7]. Ultrasonography remains the main method for the diagnosis of extra uterine pregnancy. It usually shows no uterine wall surrounding the fetus, fetal parts close to the abdominal wall, abnormal lie and/or no amniotic fluid between the placenta and the fetus [10]. In our case, bicornuate uterus with intrauterine pregnancy was considered on ultrasound examination. This may be due to the implantation of the placenta on the posterior aspect of the empty uterus and recognized as sharing the myometrium.

Intrauterine growth restriction is common in advanced abdominal pregnancies [7] in our case; the newborn was only 1.8 kg at 37 weeks of gestation and the FL to AC ratio was 24.2. These two evidences showed us the newborn had intrauterine growth restriction [11]. Intrauterine growth restriction may also be caused by the severe preeclampsia.

Advanced abdominal pregnancy with sever preeclampsia is reported rarely [12, 13]. This may be due to under reporting or due to the rare nature of advanced abdominal pregnancy by itself [12, 13]. Various theories have been forwarded to explain pre-eclampsia/eclampsia but basic to its occurrence is the presence of placental tissue in the maternal body and it is postulated that poor placentation resulting from inappropriate uterine spiral artery invasion may be the primary pathology [7, 13, 14]. This may explain the occurrence of severe pre-eclampsia in our case. It is very difficult to find causal relationship between the two conditions but from this case we can clearly understand the role of the endometrial cavity in development of preeclampsia may be not significant [12].

The most important issue in managing advanced abdominal pregnancy is the placental management. The massive hemorrhage that often occurs with surgery is related to the lack of constriction of the blood vessels after placental separation [9, 14]. The parietal peritoneum, mesentery and bowel are the usual sites where the placenta attached firmly, and there is no bleeding if it is left untouched [14]. In such cases the umbilical cord should be ligated close to the placenta, excess membranes trimmed off and the abdomen closed with drainage [14, 15]. Sometimes, the placenta may separate spontaneously simulating an abruption, but the conditions in which hemorrhage becomes uncontrollable is more likely to arise from failed attempts at placental removal [14]. Placental separation is not always straightforward and it may fail in up to 40% of cases [15]. The hemorrhage from the placental separation may be torrential and rapid surgical action is necessary to salvage the woman’s life [15]. Various local techniques can be used to stop bleeding in such cases. This may include, compression of the bleeding site, ligating the vascular pedicles, lavage with cold saline and local and/or systemic coagulation promoting agents (tranexamic acid, plasminogen derivatives, absorbable gelatin sponge, etc.) [15]. Repair of placental lacerations may need to be performed [15]. The removal of the organ to which the placenta is adherent (hysterectomy and/or salpingoophrectomy, resection of the bowel and/or bladder) maybe justified to control the hemorrhage [6, 14, 15]. Abdominal packing has been used effectively for uncontrolled hemorrhage following caesarean hysterectomy for morbidly adherent placenta, massive hemorrhage during gynecological cancer surgery and for post-partum hemorrhage [14]. However, we found only a single case report wherein it has been used to control hemorrhage in secondary abdominal pregnancy. As a last resort, the abdomen may be packed tight with abdominal packs and closed partially. The packs can be removed after 48 h or sooner if directed by hemodynamic instability [14, 15]. Alternative options for placental management includes methotrexate therapy and uterine artery embolization. Arterial embolization performed pre-operatively minimizes blood loss [16]. Placental vascular embolization facilitates resorption of a placenta that is left in situ [16].

The only two options that can be performed in our case were either to leave the placenta in place and use methotrexate or remove it and control the hemorrhage. we preferred to remove the placenta considering its favorable location (the posterior aspect of the uterus and right broad ligament, no attachments to bowel or momentum). By removing the placenta we also can avoid the potential risks of infection and spontaneous separation [6, 14, 15]. After we removed the placenta hemostatic sutures were taken to control bleeding from posterior surface of the broad ligament. However, bleeding from the posterior aspect of uterine serosa continued and we decided to pack the area. We used membrane remnants and broad ligament to keep the pack in place. We controlled the bleeding with the above technique and the patient left operation room with stable condition.

After 24 h we removed the packs. There were no surface bleeders or heamoperitoneum. The membrane remnants trimmed off and abdomen closed. The decision whether to remove the placenta or leave it in situ should therefore be individualized following careful assessment of the implantation site [16]. Our case showed that abdominal packing is effective in selected cases.

Abdominal pregnancy with live fetus is an extremely rare condition and requires a high index of suspicion. The life-threatening complication of AAP is bleeding from the detached placental site. Endometrial cavity may not be required for development of sever preeclampsia and packing is effective in controlling bleeding in selected cases.

## Abbreviations

AAP: Advanced abdominal pregnancy
AC: Abdominal circumference
FL: Femur length
